# Geospatial Mapping of People Living With Diabetes and Hypertension in Urban Slums of Central Karnataka, India

**DOI:** 10.7759/cureus.91780

**Published:** 2025-09-07

**Authors:** Shubha Davalgi, Shalini Hurlihal, Rajeev Palegar

**Affiliations:** 1 Community Medicine, Jagadguru Jayadeva Murugarajendra (JJM) Medical College, Davangere, IND

**Keywords:** diabetes mellitus, geographic information systems, gis in health, health services accessibility, hypertension, spatial analysis, urban population

## Abstract

Background: Diabetes mellitus and hypertension continue to rise in India, particularly among socioeconomically disadvantaged urban slum populations. This study presents a geographic information system (GIS)-based approach to mapping the geospatial distribution and sociodemographic profile of affected individuals.

Objectives: To determine the geospatial distribution and sociodemographic characteristics of individuals living with diabetes mellitus and hypertension in urban slums of central Karnataka, India.

Methodology: A community-based cross-sectional survey was conducted in the field practice areas of the Urban Health Training Centre, Jagadguru Jayadeva Murugarajendra (JJM) Medical College, Davangere, in central Karnataka, from November 2024 to April 2025. Data were collected via house-to-house visits using structured questionnaires programmed on Android phones via Epicollect5 (Centre for Genomic Pathogen Surveillance, Big Data Institute, University of Oxford, Oxford, UK). Geotagging of residences was done, and GIS mapping was created using QGIS software (QGIS Development Team, Open Source Geospatial Foundation, Beaverton, OR).

Results: Among 576 individuals, 112 (19.4%) had diabetes, 174 (30.3%) had hypertension, and 290 (50.3%) had both. The majority (192, 33.3%) were elderly, with a mean age of 58 ± 6.12 years. Females comprised 55.9% (322), and most participants (187, 32.4%) had formal education up to 10th grade. A total of 345 (59.9%) belonged to the low socioeconomic class, as per the modified BG Prasad classification (updated for January 2025). Clustering was observed in specific slums.

Conclusion: GIS-based mapping enables the identification of areas with a high burden of disease, facilitating targeted public health interventions in underserved urban slum settings.

## Introduction

Urbanization has resulted in a rapid shift in lifestyle and epidemiological trends across India. This includes an alarming rise in non-communicable diseases (NCDs), particularly diabetes mellitus and hypertension. These conditions disproportionately affect the urban poor, largely due to lifestyle factors such as poor diet, physical inactivity, stress, and limited healthcare access [1-3].

Slums, which constitute the living spaces for a significant proportion of India's urban population, often lack basic amenities, including access to quality healthcare. Addressing the burden of NCDs in such communities requires innovative and data-driven approaches [4,5].

Geographic information systems (GIS) provide an effective method to spatially map and analyze disease distribution, enabling stakeholders to identify clusters and direct resources efficiently [6]. Previous studies have demonstrated the utility of GIS in mapping various diseases and healthcare access disparities in both developed and developing countries [7-9].

With this background, the current study aimed to identify the geospatial distribution and socio-demographic profile of people living with diabetes and hypertension in urban slums of Davangere, Karnataka, to facilitate improved planning of healthcare interventions.

## Materials and methods

Study design and setting

This was a community-based cross-sectional study conducted in all five urban slums that constitute the designated urban field practice area of the Department of Community Medicine of Jagadguru Jayadeva Murugarajendra (JJM) Medical College, Davangere, situated in central Karnataka, India. Since the field practice area comprises five slums, all were included in the study, and no additional sampling was undertaken. The research protocol was approved by the Institute Ethics Committee of the JJM Medical College, Davangere (JJMMC/IEC/50-2024). Data collection was conducted between November 2024 and April 2025, and the present analysis is based on the finalized dataset obtained during this period.

Inclusion criteria

Inclusion criteria included adults aged ≥30 years with a known history of diabetes mellitus and/or hypertension and residing permanently in the study area.

Exclusion criteria

Exclusion criteria included individuals who were critically ill or unable to participate in the interview due to severe physical or mental health conditions, pregnant women with pregnancy-related hypertension or gestational diabetes, which may confound the findings, and participants who declined to provide informed consent or were not available during the visits.

Data collection

Trained medical interns, accompanied by medico-social workers and local Accredited Social Health Activists (ASHAs), conducted household visits under faculty supervision. A structured questionnaire was administered using Epicollect5, a free and user-friendly mobile data collection platform developed by the Centre for Genomic Pathogen Surveillance (CGPS) at the Big Data Institute, University of Oxford, Oxford, UK. Households with eligible individuals were identified with the assistance of local public health workers (ASHAs) and were visited after prior intimation. Each household was visited at least three times to collect data from all eligible and consenting individuals. After explaining the purpose of the visit and obtaining written informed consent, data were collected using mobile phones.

Study tool

The data were collected using a structured questionnaire (Appendix 1), based on the WHO STEPS instrument and adapted to the local context, which was developed by the authors specifically for this study based on a literature review and field expertise. It comprised several sections.

Sociodemographic Profile

Age, sex, education level, occupation, and socioeconomic status. The Prasad’s social classification, revised for January 2025 using the real-time update tool, was used for the socioeconomic status classification [10].

Medical History

Self-reported diagnosis of type 2 diabetes, hypertension, and any other known chronic conditions, along with treatment history.

Anthropometric Details

The height and weight were measured, which were used to calculate BMI. Body mass index (BMI) was classified according to the WHO Asian criteria: underweight (<18.5 kg/m²), normal (18.5-22.9 kg/m²), overweight (23-24.9 kg/m²), and obese (≥25 kg/m²).

Household Location Data

Global Positioning System (GPS) coordinates were recorded using the Epicollect5 mobile application at the point of data entry. The questionnaire was pilot-tested in a neighboring urban slum area (n = 20) and modified based on feedback to improve clarity and cultural appropriateness. Data were collected by trained personnel to ensure accuracy and participant comfort. All eligible adults within the selected households were included in the survey. This was done to ensure comprehensive geospatial mapping of diabetes and/or hypertension, as excluding eligible individuals would have underestimated the true burden within the community.

Data analysis

Data collected via Epicollect5 were exported into Microsoft Excel 2016 (Microsoft Corp., Redmond, WA) and analyzed using SPSS version 26.0 (IBM Corp., Armonk, NY) and QGIS version 3.22 (QGIS Development Team, Open Source Geospatial Foundation, Beaverton, OR) using the WGS 84/UTM Zone 43N projection (EPSG:32643), appropriate for the Davangere region. Descriptive statistics were used to summarize sociodemographic variables, disease prevalence, and comorbidity patterns. Continuous variables (e.g., age) were expressed as mean ± standard deviation, while categorical variables were reported as frequencies and percentages. Bivariate analysis using chi-square tests was performed to assess the association between disease status (diabetes, hypertension, or both) and demographic variables like age group, sex, education, and socioeconomic status. Spatial analysis was conducted using QGIS (version 3.22). GPS coordinates were used to plot individual households on a base map of the urban slum area. Point distribution maps were generated for individuals with diabetes, hypertension, and both. Kernel density estimation (KDE) was applied using the Quartic (biweight) kernel function, with a fixed bandwidth of 100 meters based on the average inter-household distance, and an output cell size of 10 meters to balance resolution and processing efficiency. Heatmaps were generated to visualize spatial patterns and guide interpretation. Cluster identification was done using visual inspection of KDE contours, which were then cross-referenced with known health facility locations to assess accessibility gaps. The results from statistical and spatial analyses were triangulated to understand the social and geographic determinants of disease burden and to identify priority areas for targeted intervention.

## Results

A total of 576 individuals diagnosed with diabetes and/or hypertension were included in the study. Among them, 112 (19.4%) had type 2 diabetes alone, 174 (30.2%) had hypertension alone, and 290 (50.3%) were living with both conditions. The mean age of the participants was 58 ± 6.12 years, and the majority of the participants (192, 33.3%) were elderly (≥60 years). Females comprised a slightly higher proportion (322, 55.9%) compared to males (254, 44.1%) (Table 1).

**Table 1:** Gender-wise distribution of sociodemographic characteristics of the study participants. Data were collected using the structured questionnaire developed by the authors. Credits: Davalgi S, Hurlihal S, and Palegar R.

Age group (years)	Males, n (%)	Females, n (%)	Total, n (%)
30–39	18 (7.1%)	24 (7.5%)	42 (7.3%)
40–49	47 (18.5%)	56 (17.4%)	103 (17.9%)
50–59	64 (25.2%)	71 (22.0%)	135 (23.4%)
60–69	82 (32.3%)	98 (30.4%)	180 (31.3%)
≥70	43 (16.9%)	73 (22.7%)	116 (20.1%)
Total	254 (100%)	322 (100%)	576 (100%)
Education level	Males, n (%)	Females, n (%)	Total, n (%)
Up to 10th standard	81 (31.9%)	106 (32.9%)	187 (32.4%)
Above 10th standard	58 (22.8%)	41 (12.7%)	99 (17.2%)
Illiterate	115 (45.3%)	175 (54.3%)	290 (50.3%)
Total	254 (100%)	322 (100%)	576 (100%)
Socioeconomic class	Males, n (%)	Females, n (%)	Total, n (%)
Class I–III	63 (24.8%)	50 (15.5%)	113 (19.6%)
Class IV	81 (31.9%)	37 (11.5%)	118 (20.5%)
Class V	110 (43.3%)	235 (73.0%)	345 (59.9%)
Total	254 (100%)	322 (100%)	576 (100%)
Occupation status	Males, n (%)	Females, n (%)	Total, n (%)
Working	118 (46.5%)	92 (28.6%)	210 (36.5%)
Retired/not working	136 (53.5%)	230 (71.4%)	366 (63.5%)
Total	254 (100%)	322 (100%)	576 (100%)

Sociodemographic profile

Analysis of age distribution showed the highest proportion of individuals (78, 30.5%) were in the 50-59 years age group, followed by 60-69 years. However, chi-square analysis revealed no statistically significant difference in age distribution between males and females (χ² = 2.31, p = 0.678) (Table 1).

Educational attainment showed that 187 (32.4%) of the participants had completed formal education up to the 10th standard, while 122 (21.2%) were illiterate. Gender-wise analysis revealed a significant difference: 72 (28.1%) males were literate compared to only 62 (24.2%) females, while 97 (37.9%) females were illiterate compared to 25 (9.8%) males. This difference was statistically significant (χ² = 28.59, p < 0.0001) (Table 1).

Occupationally, the majority (366, 63.5%) were either retired or not engaged in active employment, with only 210 (24.6%) reporting active work status. Gender differences in occupation distribution were not significant (χ² = 1.18, p = 0.2777).

Socioeconomic classification using the modified BG Prasad scale (January 2025 update) revealed that most participants (345, 59.9%) belonged to class V (lowest strata). Gender-wise, 126 (49.2%) females were in class V compared to 31 (12.1%) males, and this difference was highly significant (χ² = 54.82, p < 0.0001) (Table 1).

Clinical profile and anthropometry

Among the 576 participants, 402 (69.8%) self-reported one or more chronic conditions. Most were under treatment; however, many reported irregular medication adherence. Mean BMI was 25.6 ± 4.8 kg/m², with 416 (72.2%) categorized as overweight or obese (BMI ≥23). A higher prevalence of elevated BMI was observed among those with both diabetes and hypertension (Tables 2, 3).

**Table 2:** Clinical profile of study participants. For clarity, the overall prevalence among surveyed subjects was as follows: diabetes (402, 69.7%) and hypertension (464, 80.5%). Percentages in the table reflect condition-specific counts without double-counting for treatment status. Data were collected using the structured questionnaire developed by the authors. CVD: cardiovascular disease; CKD: chronic kidney disease. Credits: Davalgi S, Hurlihal S, and Palegar R.

Condition	Number of individuals (n)	Percentage (%)	On regular treatment, n (%)
Type 2 diabetes mellitus (only)	112	19.4%	94 (83.9%)
Hypertension (only)	174	30.2%	142 (81.6%)
Both diabetes & hypertension	290	50.3%	236 (81.4%)
Other chronic conditions (e.g., asthma, CVD, and CKD)	68	11.8%	49 (72.1%)

**Table 3:** Anthropometric characteristics of study participants. * WHO Asian criteria. Data were collected using the structured questionnaire developed by the authors. Credits: Davalgi S, Hurlihal S, and Palegar R.

BMI category*	BMI range (kg/m²)	Number of individuals (n)	Percentage (%)
Underweight	<18.5	28	4.9%
Normal	18.5 – 22.9	132	22.9%
Overweight	23.0 – 24.9	151	26.2%
Obese class I	25.0 – 29.9	183	31.8%
Obese class II	≥30.0	82	14.2%
Total	–	576	100%

Association between sociodemographic variables and disease status

The bivariate analysis revealed significant associations between select sociodemographic variables and disease status among the study participants. Age group showed a strong and statistically significant relationship with disease categories (χ² = 24.36, p < 0.001). Educational status was found to be significantly associated with disease status (χ² = 16.21, p = 0.012); a larger proportion of illiterate individuals were found among those with hypertension (79, 45.4%) and diabetes (43, 38.4%), whereas participants educated up to 10th standard made up a higher share of those with both conditions (190, 65.5%). Socioeconomic status, as per the modified BG Prasad classification, also demonstrated a significant association (χ² = 21.84, p = 0.001). The majority of individuals across all disease categories belonged to class V, the lowest socioeconomic tier, comprising 70 (62.5%) of diabetic, 102 (58.6%) of hypertensive, and 173 (59.7%) of participants with both conditions. These findings underscore the disproportionate burden of NCDs among the elderly, less educated, and socioeconomically disadvantaged populations living in urban slums (Table 4).

**Table 4:** Association between sociodemographic variables and disease status among study participants (n = 576). * Statistically significant at p < 0.05. Data were collected using the structured questionnaire developed by the authors. Credits: Davalgi S, Hurlihal S, and Palegar R.

Variable	Diabetes (n = 112)	Hypertension (n = 174)	Both (n = 290)	Chi-square (χ²)	p-value
Age group (years)					
<50	14 (12.5%)	25 (14.4%)	38 (13.1%)		
50–59	32 (28.6%)	41 (23.6%)	78 (26.9%)		
≥60	66 (58.9%)	108 (62.1%)	174 (60.0%)	24.36	<0.001*
Sex					
Male	50 (44.6%)	78 (44.8%)	126 (43.4%)		
Female	62 (55.4%)	96 (55.2%)	164 (56.6%)	0.73	0.694
Education					
Illiterate	43 (38.4%)	79 (45.4%)	100 (34.5%)		
Upto 10th standard	69 (61.6%)	95 (54.6%)	190 (65.5%)	16.21	0.012*
Socioeconomic class					
Class V	70 (62.5%)	102 (58.6%)	173 (59.7%)		
Class IV and above	42 (37.5%)	72 (41.4%)	117 (40.3%)	21.84	0.001*

Spatial distribution and GIS mapping

**Figure 1:**
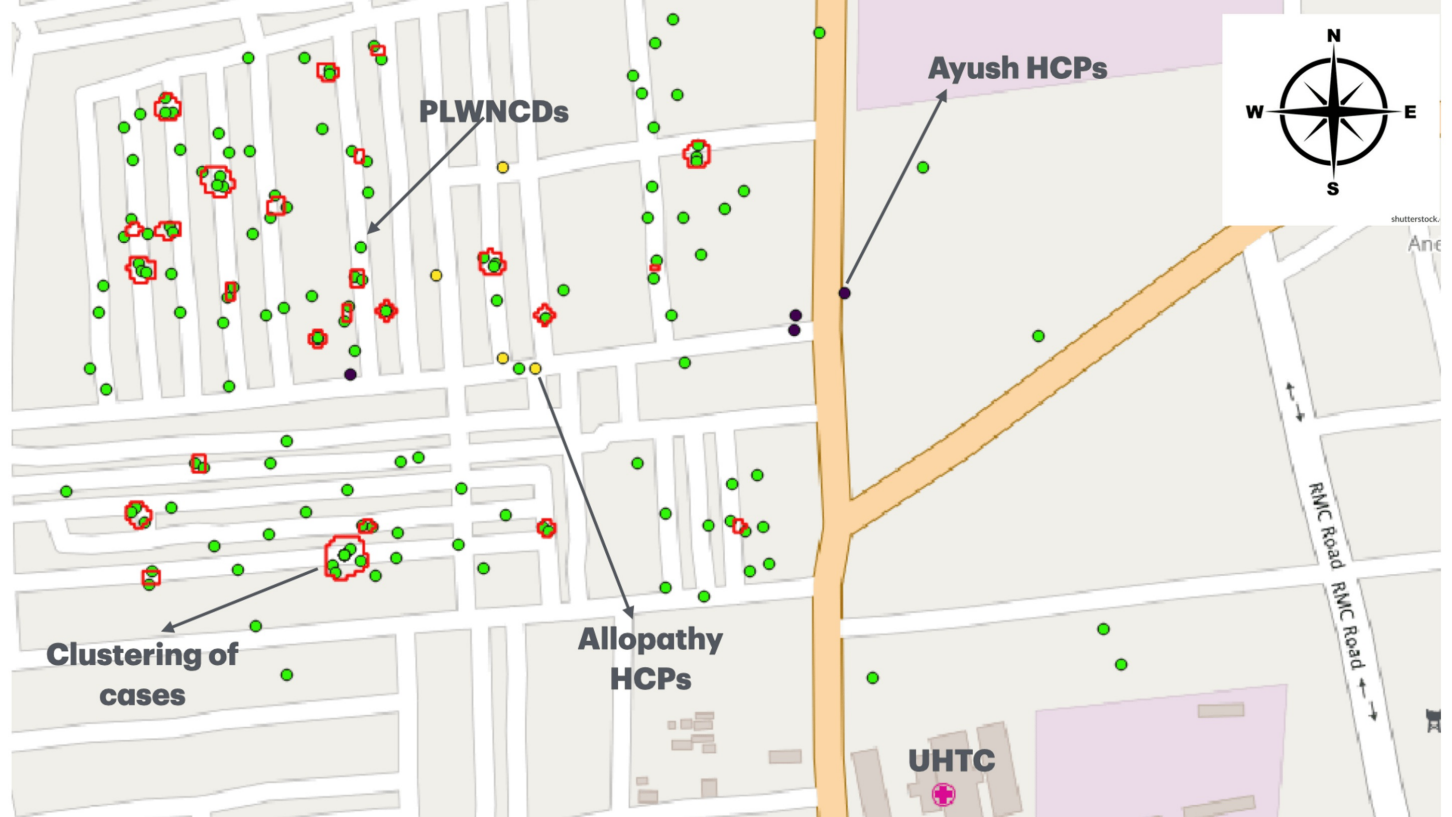
Geospatial mapping of people living with NCDs, with clustering of cases and healthcare providers in urban slums of central Karnataka using QGIS on Open Street Map. UHTC: Urban Health Training Centre; PLWNCDs: people/study participants living with NCDs (diabetes & hypertension); HCPs: healthcare providers; Ayush: Ayurveda, Yoga & Naturopathy, Unani, Siddha, and Homeopathy; NCDs: non-communicable diseases.

Figure 1 illustrates the geographic clustering of individuals diagnosed with NCDs, specifically, type 2 diabetes mellitus, hypertension, or both, within the urban field practice area. Each green dot represents a study participant, while red circles indicate spatial clusters where a higher concentration of people living with non-communicable diseases (PLWNCDs) was observed.

The GIS-based map also depicts the location of various healthcare providers, including Urban Health Training Centre (UHTC), private clinics, and community-based practitioners. While some high-burden clusters were within 1-2 km proximity to healthcare services, others were more isolated, underscoring geographical disparity in service accessibility (Figure 1).

These clusters point to potential NCD hotspots in the community. Healthcare provider (HCP) locations are also mapped. Purple dots represent AYUSH (Ayurveda, Yoga & Naturopathy, Unani, Siddha, and Homeopathy) healthcare providers, yellow dots indicate allopathy healthcare providers, and the pink cross symbol denotes the location of the UHTC, the primary health facility catering to the study population (Figure 1).

The spatial distribution reveals a visible concentration of NCD cases in specific pockets, particularly in densely populated residential areas on the left half of the map. These zones may require prioritized community-based interventions. The proximity of healthcare providers to clusters varies; while some clusters are close to the UHTC or HCPs, others are relatively underserved, highlighting potential gaps in access to care (Figure 1).

This GIS-based mapping emphasizes the utility of geospatial analysis in visualizing disease burden and aligning healthcare service delivery with areas of greatest need.

## Discussion

This study provides a comprehensive assessment of the sociodemographic and clinical profile of PLWNCDs in an urban slum setting, coupled with a geospatial visualization of disease clustering and the distribution of healthcare providers. The prevalence and spatial patterns observed underscore the urgent need for location-sensitive approaches to chronic disease management in low-resource urban areas. Our results revealed that diabetes and hypertension are highly prevalent among older adults, with a greater burden among females and those from lower socioeconomic classes. Overweight and obesity also emerged as important correlates in our descriptive analysis, reinforcing their critical role as modifiable risk factors in the causal pathway of metabolic syndrome, diabetes, and hypertension. While multivariate regression could provide insights into the independent effects of obesity and other sociodemographic factors, such modeling was beyond the descriptive scope of the present study. The future research would extend the current work with multivariate modeling to assess the independent contributions of sociodemographic characteristics and overweight/obesity to clustering patterns.

Nearly half of our participants reported both conditions, illustrating the syndemic nature of NCDs in urban poverty contexts. Distinct clusters of PLWNCDs were identified, and spatial analysis demonstrated mismatches between high-burden zones and available healthcare providers.

These findings are consistent with evidence from other Indian urban slum studies, including cross-sectional research from Punjab [7], Puducherry [8], and Delhi [9], which similarly reported a disproportionately high NCD burden in socioeconomically marginalized urban communities. Prior Indian studies have also documented NCD clustering through GPS-tagged data and community-based screening [11,12]. Our contribution builds on this work by combining disease mapping with the location of healthcare providers, offering an integrated view of both disease burden and service utilization, findings that echo qualitative research from Mumbai slums, where care-seeking pathways were found to be fragmented and geographically constrained [13].

At a global level, our results mirror trends highlighted in studies covering urban slums across South Asia, Europe, and Latin America [10-14]. That review noted wide variability in prevalence between and within countries but consistently identified higher NCD risk among slum residents compared to rural counterparts. Our findings also align with studies from Dhaka, Bangladesh, where geospatial mapping of hypertension and diabetes prevalence guided spatial targeting of local health interventions [14]. Similarly, the Warwick Centre for Global Health has emphasized the value of hyperlocal mapping in informing strategic placement of health infrastructure and outreach services in urban informal settlements [15].

Common across these studies, including ours, is the observation that disease burden in slum communities is not evenly distributed. Gender disparities, educational attainment, and income levels strongly correlate with disease prevalence, and our bivariate analysis affirms significant associations between these demographic factors and disease status. Furthermore, the co-existence of diabetes and hypertension in nearly half of our sample illustrates the syndemic nature of NCDs in urban poverty contexts.

Our GIS analysis visualizes these findings in a way that can be directly actionable for policymakers and program managers. The identified clusters represent ideal locations for targeted screening programs, mobile health clinics, and community outreach. Additionally, the proximity mapping of allopathy and AYUSH practitioners enables more efficient referral linkages and potential public-private collaborations for chronic disease care.

This study has several notable strengths. One of the key strengths was the use of real-time geotagging through the Epicollect5 platform, which enabled precise capture of participants' residential locations. This allowed for spatial mapping and identification of disease clusters. The study design also included a comprehensive survey that integrated demographic, clinical, and spatial data using three major GIS components, offering a multidimensional understanding of the distribution and determinants of non-communicable diseases in the urban slum setting. Systematic quality checks were conducted throughout the data collection process, including random spot-checks by supervisors, periodic validation of entries in Epicollect5, and cross-verification of selected participant responses to ensure data accuracy and consistency. Robust field data collection processes further enhanced the reliability and completeness of the dataset.

However, the study also has certain limitations. Being cross-sectional in nature, it does not allow for assessment of temporal trends or causality between risk factors and disease status. Furthermore, the true burden of NCDs may have been underestimated, as undiagnosed cases were not captured, and only those with a known diagnosis were included. In addition, reliance on self-reported diagnoses without biochemical verification introduces the possibility of misclassification and may affect the accuracy of prevalence estimates. While geographic clustering of cases was described visually and narratively, formal geostatistical modeling techniques such as spatial autocorrelation or hotspot analysis were not employed. Lastly, the study did not assess cost-related or accessibility-related metrics of nearby healthcare facilities, which are important contextual factors influencing care-seeking behavior and disease management in slum populations.

## Conclusions

This study highlights the substantial burden of NCDs, particularly diabetes and hypertension, among residents of urban slums in Davangere. A significant proportion of affected individuals were elderly, female, and from the lowest socioeconomic strata, underscoring the intersection of age, gender, and poverty as key determinants of NCD vulnerability in these settings. By employing GIS tools, we identified distinct spatial clusters of individuals living with diabetes and/or hypertension, and mapped them against the availability of healthcare providers. These hyperlocal patterns revealed zones of high disease density that were either underserved or had limited proximity to formal health facilities.

Our findings emphasize the critical role of spatial epidemiology in public health planning. The ability to visualize geographic hotspots of disease prevalence and service gaps enables more effective, data-driven decisions for targeted interventions. The GIS outputs provide directly actionable insights: mobile health units and screening camps can be strategically positioned within the identified high-density clusters, thereby maximizing reach and efficiency. Furthermore, strengthening referral linkages between these hotspots and nearby healthcare providers can bridge existing service gaps. The methodology adopted, integrating demographic, clinical, and geospatial data, offers a scalable and replicable model for other low-resource urban contexts across India and globally. In conclusion, this study demonstrates that leveraging GIS not only helps visualize the NCD burden but also translates into precise, evidence-based policy guidance. Embedding such spatial insights into routine decision-making can make NCD prevention and control more equitable, accessible, and impactful for vulnerable urban slum populations.

## Appendices

Appendix 1: Community-based NCD screening questionnaire

**Table 5:** Community-based NCD screening questionnaire. NCD: non-communicable diseases; GIS: geographic information system; BP: blood pressure; PHC: primary health center; CHC: community health center; ASHA: Accredited Social Health Activist; ANM: auxiliary nurse midwife; NGO: non-governmental organization; AYUSH: Ayurveda, Yoga & Naturopathy, Unani, Siddha, and Homeopathy. Credits: Davalgi S, Hurlihal S, and Palegar R.

Section	Item	Question/measurement	Response options
A. Demographic details	1	Name of the participant	_________________________________
	2	Age (in completed years)	____________
	3	Sex	☐ Male ☐ Female ☐ Other
	4	Mobile number	_______________________
	5	Educational status	☐ Illiterate ☐ Up to 10th Std. ☐ Above 10th Std.
	6	Occupation	_________________________
	7	Socioeconomic class (Modified BG Prasad Scale – January 2025)	☐ I ☐ II ☐ III ☐ IV ☐ V
B. Clinical history	1	Known diagnosis of	☐ Diabetes mellitus ☐ Hypertension ☐ Both
	2	Year of diagnosis (approx.)	Diabetes: _______ Hypertension: _______
	3	Currently on treatment	☐ Yes ☐ No
	4	Type of treatment (if yes)	☐ Allopathic ☐ AYUSH ☐ Home Remedies ☐ Mixed
	5	Frequency of follow-up	☐ Regular (Monthly) ☐ Occasionally ☐ Rarely ☐ Never
	6	Place of diagnosis	☐ Govt. Hospital ☐ Private Clinic ☐ Health Camp ☐ Other: __________
C. Anthropometry	1	Height (cm)	__________
	2	Weight (kg)	__________
	3	BMI (to be calculated)	__________
	4	Waist circumference (cm)	__________
	5	Hip circumference (cm)	__________
	6	Waist-hip ratio (to be calculated)	__________
D. Health-seeking behavior	1	Place usually sought for diabetes/hypertension care	☐ Govt. Hospital ☐ PHC/CHC ☐ Private clinic ☐ Pharmacy ☐ Traditional healer ☐ None
	2	Main reason for choosing the facility	☐ Free care ☐ Near home ☐ Trusted ☐ Referred ☐ Other
	3	Undergoes regular BP/sugar checkups	☐ Yes ☐ No
	4	Ever hospitalized for related complications	☐ Yes ☐ No
E. Health education & awareness	1	Aware of lifestyle modifications	☐ Yes ☐ No
	2	Source of information	☐ Doctor ☐ ASHA/ANM ☐ Media ☐ Family/friends ☐ Other
	3	Aware of government schemes for free medicines/check-ups	☐ Yes ☐ No
	4	Received counseling in the last 6 months	☐ Yes ☐ No
F. GIS & location data	1	Household location	Latitude: _______ Longitude: _______ Address/Landmark: ___________________ Ward/Slum Name: ___________________
	2	Nearest healthcare facility (within 1 km)	Name: __________ Type: ☐ Government ☐ Private ☐ NGO System: ☐ Allopathy ☐ AYUSH (☐ Ayurveda ☐ Unani ☐ Siddha ☐ Homeopathy) Distance: ☐ <500 m ☐ 500–1000 m ☐ >1000 m Travel Time (min): _______ Mode: ☐ Walking ☐ Bicycle ☐ Auto-rickshaw ☐ Bus ☐ Other: _______ Aware of this facility: ☐ Yes ☐ No Ever used this facility: ☐ Yes ☐ No
